# Predictive factors for pacemaker requirement after transcatheter aortic valve implantation

**DOI:** 10.1186/1471-2261-12-87

**Published:** 2012-10-04

**Authors:** Ibrahim Akin, Stephan Kische, Lylia Paranskaya, Henrik Schneider, Tim C Rehders, Ulrich Trautwein, Gökmen Turan, Dietmar Bänsch, Olga Thiele, Dimitar Divchev, Ilkay Bozdag-Turan, Jasmin Ortak, Gunther Kundt, Christoph A Nienaber, Hüseyin Ince

**Affiliations:** 1 Heart Center Rostock, Department of Internal Medicine I, University Hospital Rostock, Rostock School of Medicine, Ernst-Heydemann-Str. 6, Rostock, 18057, Germany; 2 Institute for Biostatistics and Information in Medicine and Ageing Research, University Hospital Rostock, Rostock School of Medicine, Ernst-Heydemann-Str. 6, Rostock, 18057, Germany

**Keywords:** TAVI, AV block, Left bundle branch block, Pacemaker, His bundle

## Abstract

**Background:**

Transcatheter aortic valve implantation (TAVI) has been established as a treatment option for inoperable patients with symptomatic aortic valve stenosis. However, patients suffer frequently from conduction disturbances after TAVI.

**Methods:**

Baseline, procedural as well as surface and intracardiac ECG parameters were evaluated for patients treated with TAVI and a comparison between patients requiring pacemaker with those not suffering from relevant conduction disorders were done.

**Results:**

TAVI was successfully in all patients (n=45). Baseline surface and intracardiac ECG recording revealed longer PQ (197.1±51.2 msec versus 154.1±32.1 msec; p<0.001), longer AH (153.6±43.4 msec versus 116.1±31.2 msec; p<0.001) and HV interval (81.7±17.8 msec versus 56.8±8.5 msec; p<0.001) in patients with need for a pacemaker (n=23) versus control group (n=22); furthermore, 7-day follow-up analysis showed a higher prevalence of new left bundle branch block (LBBB) (87.0% versus 31.9%; p<0.001). Multivariate analysis revealed that only new LBBB, QRS duration >120 msec and a PQ interval >200 msec immediately (within 60 minutes) after implantation of the aortic valve were predictors for high-grade (type II second-degree and third-degree) AV block. Other clinical parameters as well as baseline electrocardiographic parameters had no impact on critical conduction delay.

**Conclusion:**

Cardiac conduction disturbances are common after TAVI. The need for pacing after TAVI is predictable by surface ECG evaluation immediately (within 60 minutes) after the procedure.

## Background

Transcatheter aortic valve implantation (TAVI) has emerged as an alternative to open surgery in patients with symptomatic aortic valve stenosis considered at high risk or unsuitable for thoracotomy [1,2]. Atrioventricular (AV) block with subsequent pacemaker requirement was described in 6% of cases after surgical aortic valve replacement, but varies after TAVI between 5.7% and 42.5%, while new left bundle branch block (LBBB) occurs in up to 50-70% [2-6]. Moreover, early prediction of pacemaker requirement after TAVI would be of potential benefit by shortening both postoperative monitoring and hospitalization. We compared both clinical parameters as well as surface and intracardiac ECG recordings prior and after TAVI and analyzed differences in patients eventually requiring permanent pacing after TAVI for relevant conduction delay.

## Methods

### Patients

In our retrospective analysis, between January 2007 and January 2008 45 patients without previously known pacemaker who underwent TAVI using the third-generation percutaneous self-expanding CoreValve prosthesis (Medtronic Inc; Minneapolis, USA) were identified for this analysis. The criteria for inclusion and exclusion to the TAVI procedure have been described elsewhere [1-4]. In brief, patients were included with echocardiographic measurements of severe native valvular stenosis and an area < 1 cm^2^, or < 0.6 cm^2^/m^2^ regardless of adjunct valvular regurgitation; a diameter of basal orifice of the stenosed valve between 20 and 27 mm; and a diameter at the sinutubular junction ≤ 43 mm. Most importantly, all patients were considered unfit for open surgery with an EuroSCORE ≥ 20% [7]. TAVI was suggested in agreement between a cardiac surgeon and both, a clinical and interventional cardiologist; patient`s or referring physician`s preference was not relevant [8,9]. Treatment strategy was in compliance with the Helsinki Declaration. Our local ethics committee approved this treatment strategy. Patients gave informed consent prior TAVI. Pacemaker implantation at follow-up was considered indicated in case of complete AV-block, type II second-degree AV block, and in presence of new LBBB in combination with HV prolongation ≥ 75 msec. We compared patients requiring a new pacemaker within 7 days with those not requiring any pacemaker and no significant conduction delay after TAVI.

### Procedure

Details of the implantation technique have been described elsewhere [1-4]. In brief, all procedures were performed in an interventional hybrid suite under general anaesthesia to assure stable hemodynamic and minimize patient movement. TAVI was performed via femoral access under fluoroscopy; the aortic valve was initially dilated using a standard valvuloplasty balloon with a nominal diameter similar to the aortic valve and followed by CoreValve (Medtronic Inc.) insertion [1,3-6]. In each patient, prior to TAVI and aside a right ventricular bipolar pacing lead (Pacel™, St. Jude Medical, St Paul, MN, USA), a 6F quadripole electrode catheter with ring electrodes (5-mm interpole distance) (Webster D™, Biosense Webster, Diamond, USA) was introduced and advanced to the His bundle. A 6F quadripolar electrode catheter (Soloist™, Medtronic, Minneapolis, MN, USA) was advanced to the right atrium and another to the right ventricle to record a bipolar electrogram and for programmed atrial and ventricular stimulation. The access sites for all electrophysiologic electrode catheters were the femoral veins. With such instrumentation the sinus node recovery time (SNRT), corrected sinus node recovery time (c-SNRT), antegrade and retrograde effective refractory period (ERP) of the AV node, as well as intracardiac conduction times (atrium to His and His to ventricle time; AH and HV interval) were assessed. The rational to measure SNRT was the observations of sinus node arrest in a single patient receiving a CoreValve prior this study. Thus, we wanted to avoid oversee sinus node pathology in this elderly patients suffering from a high comorbidity index. All measurements were done on an Axiom Sensis™ (Siemens, Erlangen, Germany) electrophysiology workstation prior to the valve implantation, immediately after valve implantation and at a follow-up of 7 days. The AV nodal ERP was measured by introducing a single extrastimulus (S2) after a drive train of 8 stimuli at a fixed rate (S1) (600ms), at which time the S1-S2 interval is decreased until the S2 impulse does not conduct to the His bundle. To assess conduction disorders, patients were attached to uninterrupted ECG monitoring using the Philips monitoring system (IntelliVue™, Best, The Netherlands) that is installed at our ICU/IMC unit. All patients were prophylactically given a temporary pacemaker via the existing femoral venous access; with VVI mode the active pacing was 60 bpm for at least 24 hours.

### Statistical methods

All data were stored and analysed using SPSS statistical package 17.0 (Chicago, Illinois, USA). The statistics computed included mean and standard deviations of continuous variables and are presented as means ± SD, frequencies and relative frequencies of categorical factors. The Fisher exact test and chi^2^-test were used to compare proportions. A normal distribution of differences was confirmed by the Kolmogorov-Smirnov test; in presence of normally distributed data the t-test was used while with non-normal distribution the Mann–Whitney U-test was applied. The logistic regression model was used to assess the interdependence of high-grade AV block (type II second-degree and third-degree) at 7 day follow-up from prognostic factors. First, univariate analyses were performed to reveal unadjusted significant associations between prognostic variables and high-grade AV block. Thereafter, variables yielding p-values ≤ 0.25 with univariate analysis were entered in the multivariate model to assess adjusted associations between outcomes and covariates which were univariate and of borderline significance. All p-values resulted from two-sided statistical tests and ≤ 0.05 was considered to be significant.

## Results

The analysis included 18 men and 27 women at a mean age of 81.5±6.8years with severe aortic valve stenosis and no previously known pacemaker. All patients had qualified for TAVI following recent recommendations [8,9]. We compared patients requiring new pacemaker implantation during hospital stay (n=23) with those not suffering from relevant conduction delays (n=22) in the attempt to elucidate predictors for pacing. Smokers (34.8% versus 18.2%; p=0.024) as well as patients suffering from angina, with ischemic heart disease, previous stroke or peripheral arterial disease were more frequent in the pacemaker group. Moreover, logistic EuroSCORE operative mortality estimate was higher in patients requiring a pacemaker compared to the non-pacemaker group (24.0±14.6% versus 18.4±19.2%; p=0.034). Echocardiographic measurements on aortic stenosis severity and left ventricular function as well as intraoperative data revealed similar findings in both groups (Table 1).

**Table 1:** Baseline characteristics of study population stratified in pacemaker (PM) and non pacemaker group (N-PM)

	**PM (n=23)**	**N-PM (n=22)**	**p**
Clinical parameters			
Male, n (%)	10 (43.5)	8 (36.4)	0.763
Age (yrs)	80.7 ± 5.94	81.55 ± 7.90	0.684
Body mass index (kg/m^2^)	26.9 ± 3.9	26.8 ± 4.0	0.883
Hypertension, n (%)	22 (95.7)	19 (86.4)	0.346
Smoker, n (%)	8 (34.8)	4 (18.2)	0.024
Diabetes mellitus, n (%)	10 (43.5)	7 (31.8)	0.542
Creatinine (μmol/l)	151.2 ± 157.4	114.3 ± 51.6	0.372
Renal insufficiency (creatinine level > 1.5mg/dl), n (%)	14 (60.9)	11 (50)	0.554
Chronic obstructive pulmonary disease, n (%)	4 (17.1)	4 (18.2)	0.855
New York Heart Association functional class			
I	0 (0)	0 (0)	0.263
II	0 (0)	2 (9.1)	
III	15 (65.2)	15 (68.2)	
IV	8 (34.8)	5 (22.7)	
Logistic EuroSCORE (%)	24.0 ± 14.6	18.4 ± 19.2	0.034
Dyspnoe, n (%)	13 (56.6)	12 (54.6)	0.628
Angina, n (%)	11 (47.5)	6 (27.3)	0.022
Syncope, n (%)	3 (13.0)	8 (36.4)	0.091
Pulmonal artery pressure (mmHg)	42.8 ± 17.7	37.6 ± 19.9	0.375
Porcelain aorta, n (%)	1 (4.3)	3 (13.6)	0.346
Prior cardiac decompensation, n (%)	13 (56.5)	14 (63.7)	0.763
Ischemic heart disease, n (%)	20 (87.0)	15 (68.2)	0.017
Previous stroke, n (%)	10 (43.5)	3 (13.6)	0.047
Previous CABG, n (%)	1 (4.3)	2 (9.1)	0.838
Peripheral vessel disease, n (%)	5 (21.7)	1 (4.6)	0.002
Echocardiographic parameters			
Mean aortic valve area (cm^2^)	0.8 ± 0.2	0.7 ± 0.2	0.108
Left ventricular ejection fraction (%)	46.1 ± 10.7	50.2 ± 12.5	0.224
Peak pressure gradient (mmHg)	90.6 ± 24.1	86.6 ± 27.9	0.641
Mean pressure gradient (mmHg)	57.7 ± 15.3	55.4 ± 15.6	0.674
Aortic annulus dimension (mm)	23.30 ± 5.32	22.36 ± 6.85	0.141
Aortic bulbus dimension (mm)	29.70 ± 5.86	28.61 ± 7.46	0.893
Interventricular septal dimension (mm)	14.17 ± 3.82	13.55 ± 2.46	0.627
Aortic regurgitation grade ≥ I, n (%)	19 (82.6)	20 (90.9)	0.692
Mitral insufficiency ≥ grade I, n (%)	22 (95.7)	19 (86.4)	0.247

TAVI was successfully performed in all patients with a mean procedural time of 107.7±27.1 min and 104.9±33.3 min (p=0.725); length of stay in intensive care unit and in-hospital stay was similar in both groups. Six patients suffered from intraprocedural circulatory depression and required transient intravenous pressors; 1 patient required 1 DC shock for ventricular fibrillation during wire navigation of the calcified aortic stenosis. Post-interventional aortography revealed similar grades of aortic insufficiency (Table 2).

**Table 2:** Intraoperative data of pacemaker (PM) and non pacemaker group (N-PM)

	**PM**	**N-PM**	**p**
Procedural success, n (%)	23 (100)	22 (100)	0.999
Conversion to surgical AVR, n (%)	0 (0)	0 (0)	0.999
Intraprocedural circulatory depression, n (%)	4 (17.4)	2 (9.1)	0.635
Catecholamine therapy, n (%)	4 (17.4)	2 (9.1)	0.714
Resuscitation, n (%)	1 (4.4)	0 (0)	0.553
Defibrillation, n (%)	1 (4.4)	0 (0)	0.612
Vascular access site complication, n (%)	5 (21.8)	4 (18.2)	0.367
Contrast agent (ml)	113.3 ± 36.4	125.5 ± 60.8	0.732
Fluoroscopy time (min)	25.7 ± 1.8	23.9 ± 2.6	0.572
Procedure time (min)	107.7 ± 27.1	104.9 ± 33.3	0.725
CoreValve, n (%)			
26mm	10 (43.5)	12 (54.6)	0.112
29mm	13 (56.6)	10 (45.5)	0.386
Pre TAVI, n (%)	23 (100)	22 (100)	0.999
Post TAVI valvuloplasty, n (%)	10 (43.5)	10 (45.5)	0.566
Number of inflations, n (%)	1.08 ± 0.75	1.14 ± 0.94	0.335
Balloon diameter (mm)	21.87 ± 3.84	21.27 ± 4.44	0.432
Balloon length (mm)	55.22 ± 26.81	52.73 ± 30.27	0.613
Angiographic Aortic insufficiency (grade)	1.22 ± 0.48	1.10 ± 0.62	0.725
ICU stay (days)	2.9 ± 3.2	2.5 ± 1.8	0.686
Hospital stay (days)	13.4 ± 11.3	12.0 ± 6.5	0.237

**Figure 1:**
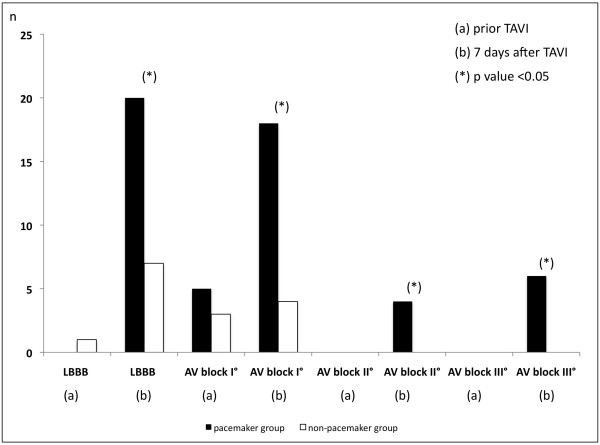
Rate of LBBB and AV block in pacemaker and non pacemaker group prior and 7 days after TAVI.

**Figure 2:**
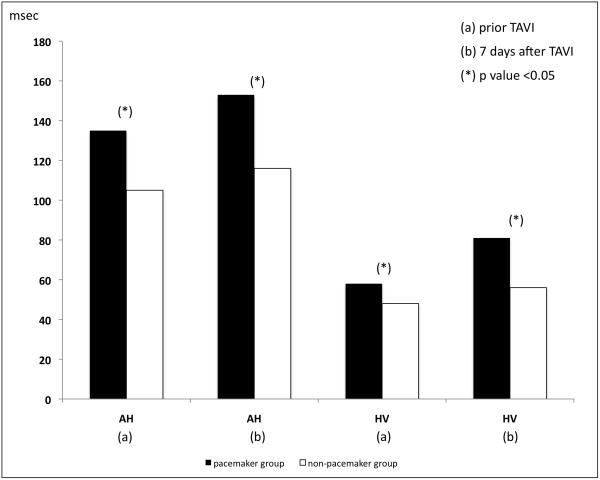
AH and HV conduction time in pacemaker and non pacemaker group prior and 7 days after TAVI.

The incidence of conduction delay requiring pacemaker implantation during 7-day follow-up after TAVI was 51.1% (n=23) due to complete AV block in 13.3% (n=6), type II second-degree AV block in 8.9% (n=4) and due to combined new LBBB with prolongation of HV interval ≥ 75 msec in 28.8% (n=13) (Table 3) (Figure 1). Baseline surface and intracardiac ECG recording revealed longer PQ interval of 197.1±51.2 msec versus 154.1±32.1 msec (p<0.001) caused by longer AH and HV intervals in the pacemaker group (Figure 2). Evaluation within 60 minutes after TAVI and at 7-day post TAVI, which was done in all patients, revealed a higher prevalence of new LBBB in pacemaker group with 87.0% versus 31.9% (p<0.001), as well as longer intervals for PQ, AH and HV interval (p<0.001). Similarly, QRS duration and antegrade and retrograde ERP were higher in pacemaker group (p<0.001) without any differences in SNRT or rates of right bundle branch block. Of 22 patients suffering from first-degree AV block 18 demonstrated prolonged HV interval (13 patients with a prolongation to ≥ 75 msec and 5 with a prolongation to < 75 msec), while 8 patients revealed a prolongation of AH interval without progression to complete heart block. Similarly, 5 patients with prolongation of HV interval to < 75 msec received no pacemaker (Table 3).

**Table 3:** Electrocardiographic characteristics

	**Prior TAVI**	**After TAVI**	**7-day follow-up**
Rhythm			
Sinus	18 (78.3)	18 (78.3)	14 (63.7)
	*20 (91.0)*	*20 (91.0)*	19 (82.7)*
Atrial fibrillation	5 (21.8)	5 (21.8)	5 (21.8)
	*2 (9.1)*	*2 (9.1)*	*5 (9.1)*
Heart rate	72.7 ± 12.6	68.5 ± 13.8	66.0 ± 16.9
	*67.5 ± 10.5*	*63.2 ± 14.4*	*65.7 ± 13.1*
Surface ECG			
PQ interval	197.1 ± 51.2	230.42 ± 40.94	243.05 ± 34.59
	154.1 ± 32.1*	167.37 ± 37.05*	168.56 ± 30.41*
QRS width	97.0 ± 15.0	139.04 ± 31.86	162.96 ± 33.59
	*95.3 ± 14.4*	110.27 ± 18.38*	120.00 ± 24.17*
QT interval	388.2 ± 29.3	409.91 ± 28.67	408.52 ± 23.22
	*398.6 ± 32.8*	*407.36 ± 32.99*	*405.14 ± 32.03*
Left-BBB	0 (0)	16 (69.6)	20 (87.0)
	*1 (4.5)*	4 (18.2)*	7 (31.9)*
Right-BBB	1 (4.35)	0 (0)	0 (0)
	*1 (4.5)*	*1 (4.6)*	*1 (4.6)*
First-degree block	5 (21.8)	8 (34.8)	18 (78.3)
	3 (13.7) *	3 (13.7)*	4 (18.2)*
Second-degree block #	0 (0)	4 (17.4)	4 (17.4)
	*0 (0)*	0 (0)*	0 (0)*
Complete block	0 (0)	4 (17.4)	6 (26.1)
	*0 (0)*	0 (0)*	0 (0)*
Intracardiac measurement			
SNRT	1086.8 ± 171.2	1093.89 ± 154.58	1122.11 ± 154.47
	*1045.6 ± 166.9*	*1050.56 ± 163.40*	*1070.59 ± 154.13*
c-SNRT	487.0 ± 54.3	492.94 ± 43.88	498.13 ± 42.72
	*462.0 ± 68.4*	*491.12 ± 42.04*	*488.59 ± 42.48*
AH interval	135.5 ± 44.7	139.32 ± 36.62	153.61 ± 43.40
	105.8 ± 31.4*	114.50 ± 38.55*	116.06 ± 31.23*
HV interval	58.5 ± 12.5	74.00 ± 13.51	81.68 ± 17.76
	48.7 ± 6.9*	55.27 ± 9.20*	56.76 ± 8.45*
Antegrade AV ERP	410.0 ± 42.6	470.00 ± 118.57	469.81 ± 45.75
	*394.4 ± 32.6*	407.78 ± 32.64*	414.71 ± 31.45*
Retrograde AV ERP	442.6 ± 38.0	470.56 ± 45.10	494.00 ± 59.33
	*420.0 ± 34.3*	436.67 ± 35.48*	441.76 ± 36.27*

* p-value <0.05.

italic : N-PM-group.

normal: PM-group.

# 2^nd^ degree (type 2) AV-block.

A multivariate analysis to identify predictors for high-grade AV block (type II second-degree and third-degree) resulting in pacemaker requirement included baseline clinical, electrocardiographic and echocardiographic parameters. With evolution to high-grade AV block as a hard endpoint in this model the univariate analysis revealed new LBBB immediately (within 60 minutes) after TAVI (OR 7.67; 95% CI 1.40-41.94; p=0.01), as well as PQ interval > 200 msec (OR 14.00; 95% CI 1.51-130.01; p=0.02) and QRS duration > 120 msec (OR 13.80; 95% CI 1.535-118.69; p=0.019) as predictors for high-grade AV block during hospital stay. Similarly, the multivariate model revealed new LBBB immediately (within 60 minutes) after TAVI (OR 24.85; 95% CI 1.57-392.57; p=0.023), as well as PQ interval > 200 msec (OR 11.37; 95% CI 1.138-97.620; p=0.02) and QRS > 120 msec (OR 14.28; 95% CI 1.50-135.88; p=0.021) as predictors for high-grade AV block. Other clinical parameters as well as baseline electrocardiographic parameters (e.g. AH interval > 100ms, HV interval > 75ms, QRS > 120ms and PQ > 200ms) had no impact to predict critical conduction delay (Tables 1, 2, 3).

## Discussion

TAVI is established as an option with reduced morbidity and mortality in high risk symptomatic patients unfit for open surgery [10-14]. Considering the proximity of aortic valve annulus to conduction system, both open surgery and TAVI may impact on both AV node and His bundle conduction.

While complete AV block was reported in 5.7%, new LBBB occurred in 18% with an association to complete AV block, syncope, and sudden cardiac arrest at long term after open surgery [15-17]. Risk factors for complete AV block after surgical aortic valve replacement include previous aortic regurgitation, myocardial infarction, pulmonary hypertension, and postoperative electrolyte imbalance [16,17], among ECG criteria right bundle branch block (RBBB) on surface ECG was the strongest predictor for pacemaker requirement [16,17].

Although previous investigations report changes in surface-ECG after TAVI [2,10-14], our group was the first to describe intracardiac conduction abnormalities for better discrimination of new ECG changes on surface-ECG, and to predict critical conduction delays [18]. In our series, complete AV block was seen in 13.3%, while 8.9% suffered from type II second-degree AV block; thus, 22.2% of patients developed an indication for permanent pacemaker implantation corroborating previous findings [10-14,19-23]. Additionally, 28.9% patients received a pacemaker for new LBBB combined with a marked prolongation of the HV interval to ≥ 75 msec for primary prevention. Such indication may be debatable, but due to the lack of data and due to the novelty of TAVI in elderly patients with several comorbidities preventive pacemaker use appears justified [24].

Jilaihawi et al. reported first that pacemaker requirement after TAVI correlates to left axis deviation at baseline, LBBB, baseline thickness of the native non-coronary cusp and to diastolic interventricular septal dimension > 17 mm [13]. Similarly, Piazza et al. revealed no prosthesis-related LBBB when the proximal end of the valve frame was positioned < 6.7 mm from the lower edge of the non-coronary cusp [10]. Degenerative calcification of the aortic and mitral annulus is probably a diffuse process, in which the cardiac conduction system is often involved and making it vulnerable to injury when exposed to mechanical compression by the nitinol frame of the CoreValve, which seems to completely expand within the first 7–10 days [14]. Differences to surgical aortic valve replacement might be due to the different techniques. In surgical approach the valve is replaced by another. Thus, the amount of conduction damage is predictable because the local trauma is nearly the same in all patients. However, in TAVI the amount of local damage is dependent of local calcification, the height of implantation in LVOT, the extend of trauma during index-procedure (balloon valvuloplasty, balloon-to-aortic annulus relation, post-TAVI dilatation) and from further aortic annulus geometry.

Our intracardiac measurements revealed that occurrence of first-degree AV block were predominantly due to prolongation of HV interval, which might be prognostically relevant [24]. We implanted pacemaker in patients with new LBBB and a HV prolongation to ≥ 75 msec in a somewhat liberal fashion in this patients with high comorbidity index. In contrast, our multivariate analysis revealed that only a PQ duration > 200 msec, a LBBB and a QRS duration > 120 msec immediately (within 60 minutes) after CoreValve implantation seem to predict critical AV conduction delay. Other baseline clinical and ECG parameters had no impact. The occurrence of above ECG findings soon after TAVI may reflect the extent of trauma from the procedure. Interestingly, the exact determination of both the amount of valve calcification and the height of implantation turned out to be non-reproducible although both parameters have been claimed to impact on conduction physiology [10,13]. For example, the Edwards SAPIEN valve, shorter and less likely to extend into the left ventricular outflow tract, is obviously associated with a lower rate of complete AV block (0-6%) [25,26]. As demonstrated by our results, we believe that regardless of favourable anatomy only the extent of trauma predict the occurrence of critical conduction delay after TAVI. According to our multivariate analysis HV prolongation ≥ 75 msec is not a predictor for pacemaker requirement. We have implanted since 2007 nearly 400 aortic valves. Our pacemaker rate was initially about 45%. This was due to the novelty of this technique and lack of information regarding the true indication for pacing. However, with further analysis of patients and improving implantation techniques (e.g. high implantation technique, no further post-TAVI balloon dilatation) our pacing rate decreased to 9-10%. Based on our data we decided not to perform routinely intracardiac measurements. The presence of a new left bundle branch block in case of normal AV conduction on surface ECG in not an indication for pacing. For clinical routine, a surface 12-lead ECG immediately (within 60 minutes) after the TAVI procedure may indicate the need for pacemaker without incremental information from intracardiac ECG recordings, the quantification of aortic valve calcification, septal thickness or height of implantation.

## Conclusion

TAVI is feasible and safe in patients unfit for open surgical aortic valve replacement. Due to the proximity of the implanted valve structure to the septum, conduction disturbances are common after TAVI requiring close surveillance at least for 7 days after TAVI. The occurrence of complete and type II second-degree AV block is predictable by a new LBBB, a PQ duration > 200 msec and QRS duration > 120 msec soon after TAVI and reflect the extent of trauma to the conduction system. Yet, the occurrence of new LBBB combined with first-degree AV block, reflecting prolongation of the HV interval, is frequent and should be subjected to further study.

### Study limitation

The present study suffers from the limitation of being a retrospective analysis. Additionally, the low number of patients may influence the results. However, our study showed that there might be no further information from intracardiac measurements as the ECG changes predominantly present as LBBB, first grade and complete AV block. The differentiation of AV block in supra-His and infra-His is not essential in these frailty patients with high comorbidity index. Thus, there will always be a somewhat “liberal” indication for pacemaker implantation. To date, there is a need for further larger scale prospective trials as well as registries with longer follow-up for predicting factors for pacemaker requirement and defining the role and duration of ECG changes.

## Abbreviations

AS: Aortic stenosis; AVA: Aortic valve area; AVR: Aortic valve replacement; c-SNRT: Corrected sinus node recovery time; ECG: Electrocardiography; ERP: Effective refractory period; ICU: Intensive care unit; IMC: Intermediate care; LBBB: Left bundle branch block; NYHA: New York Heart Association; PCI: Percutaneous coronary intervention; SNRT: Sinus node recovery time; TAVI: Transcatheter aortic valve implantation.

## Competing interests

The authors declare that they have no competing interests.

## Authors’ contribution

IA, SK, LP, HS, TCR, UT, RGT, DB, OT, DD, IBT, JO, HI, CAN participate in treating the patients during intervention / ICU and acquisition of data. IA, HI and CAN wrote the manuscript. IA and GK performed the statistical analyses. All authors read and approved the final manuscript.

## Pre-publication history

The pre-publication history for this paper can be accessed here:

http://www.biomedcentral.com/1471-2261/12/87/prepub
